# “A one-size-fits-all model is not good”?: ambivalent perceptions and experiences of African immigrant parents towards Swedish sexual and reproductive health services for young people

**DOI:** 10.1186/s13104-020-05289-7

**Published:** 2020-09-21

**Authors:** Cartrine Nancy Anyango, Faustine Kyungu Nkulu Kalengayi, Isabel Goicolea, Ida Linander

**Affiliations:** Department of Epidemiology and Global Health, Umeå University, 901 87 Umeå, Sweden

**Keywords:** Sexual and reproductive health, Youth clinics, Immigrant parents, Young people

## Abstract

**Objective:**

Parents have a key role regarding young people’s access to sexual and reproductive health services, thus their perceptions go a long way towards promoting or discouraging young people from using such services. Research has revealed that immigrant young people in Sweden access these essential services to a lesser extent than their native peers, and that they perceive their parents as unsupportive of such visits. This pilot study’s objective was to explore immigrant parents’ perceptions and experiences of the sexual and reproductive health services provided by Swedish youth clinics.

**Results:**

Two categories were developed from the data analysis: (i) Youth clinics are well-known (to some) and appreciated (to a certain extent), and (ii) Parents feel left out from youth clinics and that the clinics have taken over parental responsibility. This study presents an ambivalent scenario connected to immigrant parents’ experiences and perceptions of having neither a space nor a voice within the existing youth clinic model. Parents expressed the desire for the youth clinics to recognise their cultural backgrounds, norms, and beliefs while providing sexual and reproductive health services to their children.

## Introduction

When it comes to young people’s access to sexual and reproductive health services, parents play an important role. Thus, how parents perceive such services will affect whether they encourage their children to use them or not [1, 2]. Parents are not a homogeneous group and their perceptions of such services might be shaped by factors such as gender, class, or racialisation processes.

Several studies in the field of migration put the blame on parents when attempting to explain why immigrant young people whose parents were born in certain foreign countries access sexual and reproductive health services to a lesser extent than their native peers. They portray these parents and “their cultures” as “less progressive” in relation to issues of sexuality, especially youth sexuality [3–5].

However, we would like to point out, as other authors have previously done, that such explanations might contribute both to the further stereotyping of certain immigrant parents and to disregarding the role of racism in damaging immigrants’ trust and hindering their access to healthcare services, including young people’s sexual and reproductive health [6, 7].

In Sweden, sexual and reproductive health services for young people comprise a nationwide network of 300 youth clinics (YCs) across the country that are fully integrated into the public healthcare system to address young people’s health needs. YCs started their work in the 1970s, and nowadays deliver services through a multi-disciplinary team with special focus on sexual and reproductive health and mental health [8–10].

Our previous research, and other evaluations, show that YCs are very highly rated by young people in several domains such as fear of exposure, equity, privacy and confidentiality, non-judgemental health care providers’ attitude, quality of consultation and facility, respect and parental support to use them [11, 12]. However, the same studies have also revealed that young people born outside Scandinavia give lower ratings to certain domains of the clinics’ youth-friendliness, including those related to the extent to which they perceived their parents as likely to encourage them to visit [12]. In this qualitative pilot study, we aim to explore African immigrant parents’ perceptions and experiences of the sexual and reproductive healthcare services provided by YCs in Sweden.

## Main text

### Methods

We interviewed four parents—two men and two women, aged between 37 and 54 years, who had migrated to Sweden from four African countries and had lived in a medium-sized city for at least 10 years. Participants were recruited through support from a gatekeeper who is a researcher engaged in migrant health. The selection criteria were that the parents had sons/daughters at the age of attending YC and were aware about Swedish YCs. The potential participants were first contacted through a phone call where the study objectives were explained, and questions and doubts responded to. If afterwards they were interested in participating, an interview date was set.

We used a semi-structured interview guide focusing on the sexual and reproductive health services provided by YCs, access to those services, perceptions, experiences, challenges, and recommendations. The audio-taped interviews lasted on average 50 min.

The interviews were transcribed verbatim and analysed using qualitative content analysis [13]. CNA made the first coding, using Open Code software version 4.03 [14]. The codes and preliminary categories thus developed were then discussed with IG, and later with all the authors. Through a process of moving back and forth between these preliminary categories and the data, we finally developed two categories, which are described below.

### Results

#### Youth clinics are well-known (to some) and appreciated (to a certain extent)

This category describes parents’ ambivalent perceptions of YCs and the sexual and reproductive services they provide. Parents acknowledged and appreciated YCs, but at the same time they feared allowing their children access to some of the sexual and reproductive services that YCs provided.

The parents in this study were very much aware of the existence of YCs, which they described as “easy to find”, or as Lucy put it:

*The youth clinics are available just a call away and that’s necessary because the young people need to have somewhere where they feel safe to be able to talk about their sexuality. (Lucy)*

The quotation above also articulates parents’ perception of YCs as meeting an important need of young people: a safe space to discuss sexuality-related questions.

Parents not only knew that YCs existed and offered services related to sexuality, they also had very specific information about the diverse services that young people could get from YCs, in terms of both sexual and reproductive health and other issues, as Joab explained:

They [the youth clinic staff] explain if [young people] have a sexual problem, if they need condoms, sexual education, and if they have, for example, problems with alcohol addiction. (Joab)

However, this awareness of the existence of YCs and the positive perception of them was only one part of the story. Even though the participating parents knew about YCs and the services they provide, they also mentioned that this might not be the case for other immigrant parents. Joab, for example, described such a lack of information, or misinformation, and how it contributed to mistrust on the part of some parents:

*If YCs could cooperate with immigrant parents, I think it would increase the validity and trust in the clinics […] explain to the immigrants what the YC is doing, because the majority don’t know. (Joab)*

Thus, appreciation of the services was not without caveats. The participating parents perceived that services such as providing contraceptives, condoms, or abortion could encourage teenagers to engage in sexual activities. Such services were described as conflicting with their cultural and religious beliefs, and in this conflict, parents perceived that their religious and cultural values and roles became subordinated to those promoted by the YCs. Joab put it like this:

*Yeah, in many cases young girls just go there and have this abortion and nobody knows, and these condoms they’re just distributed free, it’s encouraging sexuality, this is not in line with our beliefs and culture. (Joab)*

#### Parents feel left out from YCs and that the clinics have taken over parental responsibility

Parents’ ambivalence towards YCs was connected to a feeling of being left out from the YC model and fearing that the YCs were taking over their parental roles. YC policy stresses that parents are not allowed to attend during consultations. This is to safeguard confidentiality and privacy during service provision and is grounded in the consideration of young people’s autonomy. While the parents valued such principles, they also struggled with the feeling of being left out from the consultation in situations when they wanted to be involved, as Caro explained:

*As a parent, I went there with my kid. Then they said you the parent can’t come inside. You must stay outside. Then I say no. Why? I want to know what people do in here. (Caro)*

Beyond being “left out” from the individual consultation, parents also spoke about feeling left out from the overall YC’s “one-size-fits-all model”. They considered that this model overlooked their values, traditions, and religious approaches, as Lucy emphasised:

*Ahaa, one model fits all isn’t good, there are societies, now we have one million immigrants from societies where sexual debut is later, where sexuality is not as openly discussed as it is here [in Sweden] and it’s problematic when you have a one model fits all. (Lucy)*

Parents regarded themselves as well equipped and able to advise their children on sexuality in line with their own cultural and religious approaches, as Caro explained:

*I am the youth clinic. I have my daughter, when a problem comes, how will it be? You [healthcare provider] won’t be there, no one will be there unless it’s me; So that’s why I say I am the youth clinic for my own family. I must protect them. (Caro).*

However, the parents also experienced that their skills were neither recognised nor valued by the YCs. In the same way as parents felt left out from the YC model, they also felt that YCs were taking over their parental role, leaving them less accountable and deprived of their parental responsibilities and rights. Lucy complained:

*She [her daughter] didn’t open up to me completely […] she said that the details of her discussion with the nurse and the people there [at the YC] she didn’t want to bring home […] and I said “are you pregnant?” and she said “mama no, that’s not how it is”. (Lucy)*

Parents expressed a desire to be involved and informed about the services provided at the YCs. As a result, they recommended that YCs should incorporate “their values”, which they believed were not always in line with “Swedish values”, as John explained:

*We have to modify it in other ways, they [healthcare providers] need to understand our children in a manner that’s valid to us and our values. We take the Swedish sexual freedom naturally, but we must talk to them [our children] about our own values. (John)*

Participants perceived that being an immigrant parent was a challenge in their interactions with YCs. They felt that YCs failed to take into consideration their way of parenting around sexuality and their cultural and religious beliefs, and that they were losing influence and control over their children in these areas. Nevertheless, this “taking over” was not only perceived as problematic. For example, Lucy recalled that in the past, in her country of birth, sexuality was discussed with other extended family members and not parents, and positively compared the role of YCs as taking over the tasks that elderly relatives used to have:

*I was also brought up in a context where I discussed my sexuality not with my mother but with my auntie and grandmother […] and so when my daughter reached puberty, most of the time I left it to the nurse because I viewed the nurse as taking that auntie role for her. (Lucy)*

## Discussion

Our findings depict an ambivalent (by ambivalence we mean that participants expressed contradictory positions in relation to many of the topics explored) scenario: parents appreciated YCs but, at the same time, were hesitant about certain services. More interestingly, this ambivalence was connected to parents’ experiences and perceptions of having neither a space nor a voice within the existing YC model.

From a cultural safety perspective, these findings suggest that YCs are not perceived as culturally safe by immigrant parents. Cultural safety includes actions that recognise and respect the cultural identity of others and takes into consideration their needs and rights. Conversely, culturally unsafe services have been described as those that “diminish, demean or disempower the cultural identity and wellbeing of an individual” [15]. Culturally safe care is important, especially in the provision of healthcare services, where there is a requirement for individuals from diverse cultures to be recognised, and their cultures, beliefs, and societal differences acknowledged [16].

Similar to other studies, our findings suggest that culture and religion play a large role in the daily activities of African immigrant families [17]. Parents in this study expressed a desire to be met halfway by the YCs: namely, to have their cultural background, values, beliefs, and norms recognised. These perceptions are in line with cultural safety principles as reported by other authors [16, 18, 19].

It is indisputable that services targeting young people should be culturally safe for them—that is, healthcare providers need to be able to adjust their behaviours and services to better meet and respond to young people’s needs and rights [16, 18–22]. The question is whether services should also be culturally safe for parents. If the criteria for cultural safety for young people and parents coincide, then making services culturally safe for both will be a win–win situation. However, parental criteria might conflict with young people’s standards, specifically in terms of autonomy in decision-making and confidentiality, as our study shows and as highlighted in our previous studies where young people rated YCs’ confidentiality and privacy high [12].

Prioritising parents’ perceptions could then deny young people the rights to access and utilise sexual and reproductive health services, threatening the youth-friendliness model to which Swedish YCs have adhered, as advocated by the World Health Organisation [1, 9].

On the flipside, ignoring parents’ perceptions might further hinder young people from accessing such services. While parents question the model, the main concern then is: if “a one-size-fits-all model is not good”, whose expectations should new models fit? Parents’, young people’s, or both? While this study poses this relevant question, further research is needed to better explore the opportunities and limitations of fulfilling parents’ expectations when designing and implementing models for young people’s sexual and reproductive health services.

## Limitations

This study included only immigrant parents with an African background. It could have been interesting to add parents from other backgrounds (Swedish, from other continents) and to contrast their perceptions with those of young people and healthcare providers. The low number of interviews is also a limitation; however, as we mentioned in the discussion, we consider that the questions brought up by this pilot study can inspire further research on this topic.

## Data Availability

The datasets used and/or analysed during the current study are available from the corresponding author on reasonable request.

## Abbreviation

YCs: Youth clinics
